# Survival Analysis and Treatment Strategies for Locally Advanced Lung Squamous Cell Carcinoma in Elderly Patients

**DOI:** 10.14740/wjon2636

**Published:** 2025-12-17

**Authors:** Zhong Fei Jia, Xian Zhe Zhao, Jing Chen Huo, Yu Xiang Wang, Jie Yang

**Affiliations:** a The Fourth Hospital of Hebei Medical University and Hebei Province Tumor Hospital, Hebei Clinical Research Center for Radiation Oncology, Shijiazhuang 050000, China

**Keywords:** Lung cancer, Chemotherapy and radiotherapy, Old age, Survival analysis, Treatment strategy

## Abstract

**Background:**

The aim of the study was to analyze the survival and treatment strategies of elderly patients with locally advanced lung squamous cell carcinoma.

**Methods:**

A retrospective analysis was conducted on the clinical and follow-up data of 278 patients with locally advanced lung squamous cell carcinoma admitted to the Fourth Hospital of Hebei Medical University from January 2012 to December 2019.

**Results:**

The 1-, 3-, 5-, and 10-year survival rates of the entire group were 86.0%, 50.6%, 40.8%, and 24.2%, respectively. There were a total of 142 cases aged 65 or elder, with 1-, 3-, 5-, and 10-year survival rates of 83.9%, 44.3%, 32.3%, and 15.6%, respectively. Among them, the progression-free survival (PFS) of the elderly group receiving radiotherapy dose ≥ 60 Gy was significantly prolonged compared to the < 60 Gy group (although P > 0.05). Comprehensive radiotherapy and chemotherapy showed a significant trend of prolonging overall survival (OS) compared to simple radiotherapy (Breslow = 0.029, log rank = 0.126). χ^2^ tests were performed for radiotherapy doses < 60 Gy and ≥ 60 Gy, PFS < 12 months and ≥ 12 months, OS < 36 months and ≥ 36 months, respectively. The proportion of PFS ≥ 12 months was significantly higher in the 60 Gy group (P = 0.043), and the proportion of OS ≥ 36 months was higher in the PFS ≥ 12 months group (P = 0.001).

**Conclusion:**

The prognosis of locally advanced unresectable lung squamous cell carcinoma is poor, especially in elderly patients. If the patient’s general condition is good, it is recommended to receive radiation therapy with a dose of ≥ 60 Gy and try comprehensive treatment with tolerable toxic side effects.

## Introduction

The most common malignant tumor in our country is lung cancer, with 1.06 million new cases in 2022. Lung cancer is also the leading cause of death among Chinese cancer patients, claiming 733,000 lives in 2022. It has become the top cause of death from malignant tumors among urban residents in China. Among all lung cancer patients, 80% to 85% are non-small cell lung cancer (NSCLC), and about one-third of NSCLC patients present with locally advanced at initial diagnosis [1]. Before a definitive diagnosis of lung cancer can be established, it is crucial to differentiate it from other conditions with similar presentations. For instance, synovial chondromatosis of the sternoclavicular joint can mimic bony metastases from lung cancer on computed tomography (CT) imaging, commonly presenting with clinical features such as joint pain, swelling, and limited range of motion [2]. Prior to confirming the diagnosis, some cases of pleural metastasis must be distinguished from pleural plaque calcification and inflammatory myofibroblastic tumor to avoid erroneous staging [3, 4].

Lung squamous cell carcinoma, due to its unique clinical manifestations, pathological features, and genetic mutation characteristics, differs significantly from lung adenocarcinoma in diagnosis and treatment. It is also explored as a separate type in clinical research. In the early stage of lung squamous cell carcinoma, there are no specific symptoms; by the time significant symptoms appear and patients seek medical attention, the disease is often advanced. Additionally, elder patients face high surgical risks, making surgery an unfeasible option, and radiotherapy or chemotherapy become crucial choices [5]. How to improve survival rate and prolong survival time for locally advanced non-surgical lung squamous cell carcinoma patients remains a key focus in clinical practice. According to the 2023 version of the Chinese Society of Clinical Oncology (CSCO) guidelines for NSCLC, concurrent chemoradiotherapy (CCRT) is the standard treatment regimen for non-surgical locally advanced lung squamous cell carcinoma. However, real-world studies have found that while radiotherapy and chemotherapy can kill tumor cells, they also cause damage to normal tissues. Moreover, elder patients with locally advanced non-surgical NSCLC often suffer from organ dysfunction and multiple underlying conditions, leading to ongoing debates about the impact of CCRT on treatment outcomes and prognosis [6]. Given this, this study focuses on the long-term efficacy and prognostic factors of CCRT for locally advanced non-surgical lung squamous cell carcinoma, particularly in elder subgroups, as well as the patterns of failure.

## Materials and Methods

### Data collection

Clinical and follow-up data were collected from 278 patients with locally advanced squamous cell lung cancer admitted to the Fourth Hospital of Hebei Medical University between January 2012 and December 2019. Patients’ pre-treatment clinical characteristics, radiotherapy fraction plan and dose, chemotherapy regimen and sequence of radiochemotherapy, sites of recurrence and metastasis, and toxic side effects of radiotherapy were recorded. The inclusion criteria for the study were patients with locally advanced squamous cell lung cancer who were not eligible for surgery or had refused surgery receiving radiotherapy, chemotherapy, and combined therapy.

### Treatment protocol

The initial treatment for enrolled patients involved a combination of radiotherapy and chemoradiotherapy. Induction chemotherapy, CCRT, and sequential chemotherapy regimens included: cisplatin/carboplatin/lodotin in combination with vinorelbine/paclitaxel/doxorubicin/etoposide, or monotherapy with paclitaxel, doxorubicin, or tegafur oral chemotherapy. The radiation therapy target area included lung tumors and metastatic lymph nodes in the mediastinum and supraclavicular regions, with a prescription dose of 36 - 75 Gy, 20 - 36 fractions, 1.8 - 2.0 Gy/fraction sessions, and 5 weekly treatments.

### Follow-up

Follow-up methods include case file review, outpatient follow-up, and telephone follow-up. Regular follow-ups after surgery were conducted, with examination items including clinical history inquiry, physical examination, chest and abdominal CT scans, head magnetic resonance imaging (MRI), positron emission tomography (PET)/CT, superficial lymph node ultrasound, bone imaging, and other radiological examinations. Recurrence and metastasis are primarily diagnosed based on CT and MRI findings, with a few cases confirmed by pathology. The follow-up deadline is July 1, 2024, with a follow-up duration ranging from 2 to 132 months, and a median follow-up time of 36.5 months (95% confidence interval (CI): 28.8 - 46.7). Five cases (1.8%) were lost to follow-up, resulting in a follow-up rate of 98.2%.

### Statistical analysis

SPSS 25.0 software was used for statistical analysis, and χ^2^ test was applied to compare categorical data. The Kaplan-Meier method was used to calculate OS, PFS1, and PFS2; Log rank method for univariate analysis; Cox model for prognostic analysis, with P < 0.05 indicating statistically significant differences. Recent efficacy was evaluated according to WHO’s RECIST criteria, and adverse reactions were assessed using the RTOG acute radiation injury grading system. Overall survival (OS) was the time from the start of treatment to death. At the end of this study follow-up, the OS for surviving patients was the last follow-up time. Progression-free survival (PFS) was the time from the start of treatment to disease progression. At the end of this study follow-up, the last follow-up time for patients without disease progression or death was PFS. PFS1 was the time from the start of treatment to the first disease progression. At the end of this study follow-up, the last follow-up time for patients without disease progression or death was PFS1. PFS2 was the time from the start of treatment to the second disease progression. At the end of this study follow-up, the last follow-up time for patients without second disease progression or death was PFS2. Local recurrence is defined as the appearance of lymph node metastasis in the supraclavicular area or mediastinum, or progression of lesions within the lungs; distant metastasis is defined as the appearance of metastatic lesions in any part of the body outside the local region; mixed metastasis is defined as cases where both local recurrence and distant metastasis coexist. Single organ metastasis is defined as metastasis to one extrathoracic organ.

This is a retrospective study that has been approved by our hospital’s ethics committee.

## Results

### Characteristics of enrolled cases and completion of treatment

A total of 278 patients with locally advanced lung squamous cell carcinoma, diagnosed pathologically as such at the Fourth Hospital of Hebei Medical University from January 2012 to December 2019, were collected along with complete clinical and follow-up data. Among them, 186 cases were moderately or highly differentiated squamous cell carcinomas, 87 cases were poorly differentiated squamous cell carcinomas, and five cases were adenosquamous carcinomas. The group consisted of 255 males and 23 females, with a median age of 64 years (range 29 - 86). There were 142 cases under 65 years old and 136 cases aged 65 or elder. Central lung cancer accounted for 204 cases, while peripheral lung cancer accounted for 74 cases. In terms of TNM staging, 106 cases were stage IIIa, 121 cases were stage IIIb, and 51 cases were stage IIIc (TNM staging according to the eighth edition of the International Society for Clinical Lung Oncology (IASLC) TNM classification) (Table 1).

**Table 1:** Clinical Characteristics of 278 Cases of Locally Advanced Lung Squamous Cell Carcinoma

Factor	Group	Case, n (%)
Gender	Male	255 (91.7%)
	Female	23 (8.3%)
Age	< 65 years	136 (48.9%)
	≥ 65 years	142 (51.1%)
Location	Central	204 (73.3%)
	Peripheral	74 (26.7%)
N stage	N0	21 (7.5%)
	N1	7 (2.5%)
	N2	165 (59.4%)
	N3	85 (30.6%)
T stage	T1b	1 (0.3%)
	T1c	28 (10.1%)
	T2a	15 (5.4%)
	T2b	66 (23.7%)
	T3	58 (20.9%)
	T4	110 (39.6%)
TNM stage	IIIa	106 (38.1%)
	IIIb	121 (43.5%)
	IIIc	51 (18.4%)
Differentiation	Medium/high low	186 (66.9%)
	Adenocarcinoma	87 (31.3%)
	Squamous	5 (1.8%)

For first-line treatment completion, 49 cases (17.6%) received CCRT followed by consolidation chemotherapy, 16 cases (5.7%) received sequential chemoradiotherapy (SCRT), 63 cases (22.6%) received only radiotherapy, and 150 cases (54.1%) received induction chemotherapy plus radiotherapy (chemotherapy) or CCRT. The median induction chemotherapy cycle was three cycles (range 1 - 6 cycles), the median concurrent chemotherapy cycle was one cycle (range 1 - 3 cycles), and the median consolidation chemotherapy cycle was two cycles (range 1 - 6 cycles). Among these, 56 cases received GP regimen, 53 cases received DP regimen, 84 cases received TP regimen, and 12 cases received EP regimen chemotherapy. Conformal radiotherapy was used in 93 cases (33.4%), and intensity-modulated radiotherapy was used in 185 cases (66.6%). A total of 269 cases completed radiotherapy (96.7%), while nine cases (3.3%) did not complete it.

On completion of first-line treatment for the elderly group (age ≥ 65 years), 44 cases (32.1%) received radiotherapy alone, 92 cases (67.9%) received combined chemoradiotherapy, including 25 cases (18.2%) with CCRT and consolidation chemotherapy, eight cases (5.8%) with SCRT, 44 cases (32.1%) with radiotherapy alone, and 60 cases (43.8%) with induction chemotherapy plus radiotherapy (chemotherapy)/CCRT. Conformal radiotherapy was used in 46 cases (33.6%), and intensity-modulated radiotherapy in 191 cases (66.4%). A total of 134 cases (96.7%) completed radiotherapy, while three cases (2.2%) did not complete it, resulting in a radiotherapy completion rate of 97.8%.

### Recent efficacy and adverse reactions: post-radiotherapy efficacy evaluation

Complete response (CR) was reported in nine cases (3.2%), partial response (PR) in 181 cases (65.1%), stable disease (SD) in 84 cases (30.2%), and progressive disease (PD) in four cases (1.4%). Radiothoracic pneumonia was reported in 84 cases, incidence 30.2%, including grade I in 62 cases (73.8%), grade II in 18 cases (21.4%), and grade III in four cases (4.7%). Radioactive esophagitis was reported in 98 cases, incidence 35.2%, including grade I in 79 cases (80.6%), grade II in 18 cases (18.3%), and grade III in one case (1.9%). In the elderly subgroup, CR + PR was achieved in 75 cases (54.7%) and SD + PD in 45.3%.

### Survival and prognosis analysis

**Figure 1:**
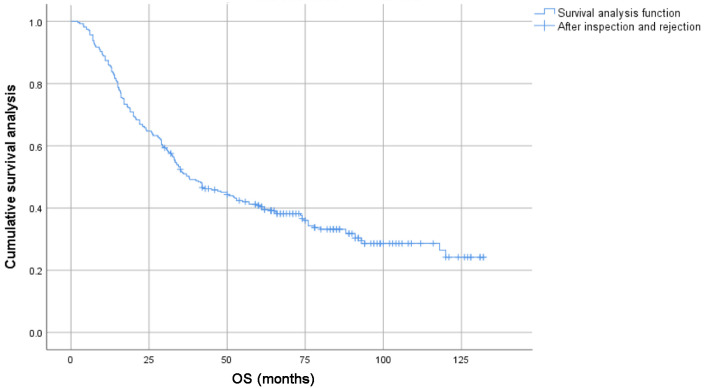
Survival curves of 278 patients with locally advanced lung squamous cell carcinoma.

**Figure 2:**
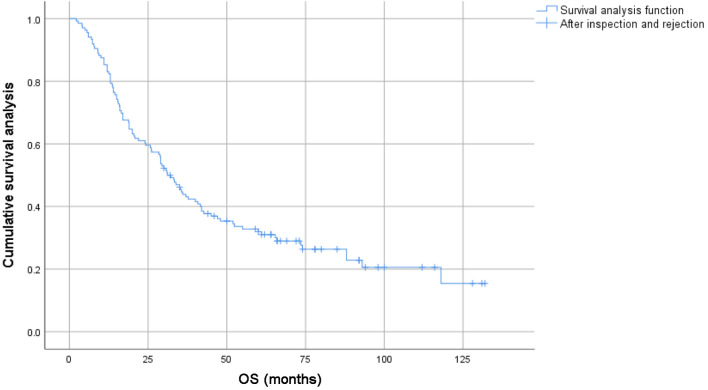
Survival curves of subgroups of 136 elderly patients with locally advanced lung squamous cell carcinoma.

The 1-, 3-, 5-y, and 10-year survival rates for the entire group were 86.0%, 50.6%, 40.8%, and 24.2%, respectively. The 1-, 3-, 5-, and 10-year PFS rates were 63.6%, 28.6%, 20.8%, and 8.3%, respectively. For patients under 65s, the 1-, 3-, 5-, and 10-year survival rates were 87.9%, 57.4%, 48.8%, and 31.5%, respectively; for patients aged 65 or elder, the 1-, 3-, 5-, and 10-year survival rates were 83.9%, 44.3%, 32.3%, and 15.6%, respectively (Figs. 1 and 2).

### Progression and metastasis after treatment

#### First-time progression sites and second-line treatment

Among 238 cases of disease progression, 108 cases (45.3%) had lung lesions progressing, 34 cases (14.2%) had mediastinal or supraclavicular lymph node metastasis, 67 cases (28.1%) had distant metastasis, and 29 cases (12.1%) had mixed metastasis. Seventy-seven cases (32.3%) had single organ metastasis. In terms of treatment regimens, 80 cases received chemotherapy, 56 cases received radiotherapy, 70 cases received combination therapy, 18 cases underwent surgery, four cases received comprehensive treatment, four cases received symptomatic treatment, five cases abandoned treatment, two cases received targeted therapy, and one case received immunotherapy.

#### Second progression sites and treatment status

Among the total of 72 cases, local progression was reported in 24 cases (33.3%), liver metastasis in six cases (8.3%), bone metastasis in 13 cases (18.1%), lung metastasis in five cases (6.9%), brain metastasis in eight cases (11.1%), mediastinal lymph node metastasis in five cases (6.9%), mixed metastasis in eight cases (11.1%), and adrenal metastasis in three cases (4.1%). After treatment, 29 cases were evaluated PR, 41 SD, and two PD.

#### Elder subgroup

Among 116 cases of disease progression, 56 cases (48.2%) had intrapulmonary lesions progressing, 12 cases (10.3%) had mediastinal or supraclavicular lymph node metastasis, 40 cases (34.4%) had distant metastasis, and eight cases (7.1%) had mixed metastasis. Single organ metastasis occurred in 42 cases (36.2%). In terms of treatment regimens, chemotherapy was received in 38 cases, radiotherapy in 30 cases, chemoradiotherapy in 31 cases, surgery in 11 cases, comprehensive treatment in one case, symptomatic treatment in three cases, and targeted therapy in two cases. Regarding second progression sites and treatment status, among the total of 27 cases, local progression was reported in 10 cases (37.0%), liver metastasis in two cases (7.4%), bone metastasis in five cases (18.5%), lung metastasis in two cases (7.4%), brain metastasis in two cases (7.4%), mediastinal lymph node metastasis in three cases (11.1%), mixed metastasis in one case (3.8%), and adrenal metastasis in two cases (7.4%).

#### OS univariate analysis

Smoking status (P = 0.012), age (P = 0.004), lesion location (P = 0.001), chemotherapy (P = 0.026), radiation pneumonitis (P = 0.041), first-line treatment efficacy (P < 0.001), and PFS stratification (P < 0.001) were associated with OS (P < 0.05) (Table 2), while gender, Eastern Cooperative Oncology Group (ECOG) score, family history of malignancy, pathological differentiation degree, T stage, N stage, TNM stage, and timing of radiotherapy were not related to OS. In multivariate Cox analysis (Table 3), age (P < 0.001), lesion location (P = 0.002), radiation pneumonitis (P = 0.048), first-line treatment efficacy stratification (P < 0.001), and PFS stratification (P < 0.001) were independent prognostic factors for OS (P < 0.05). Among these, age ≥ 65 years, central lung cancer, and radiotherapy dose < 60 Gy had poorer outcomes (P < 0.05). The combination of chemoradiotherapy did not significantly extend OS in elder patients compared to radiotherapy alone (P = 0.531).

**Table 2:** Univariate Analysis of OS in 278 Patients With Locally Advanced Lung Squamous Cell Carcinoma

Factor	Group	Case, n	OS (%)			P value
			3 years	5 years	10 years	
Smoke	No	55	67.3	55.9	33.0	0.012
	Yes	223	47.0	37.0	21.1	
Age	< 65 years	141	57.4	48.8	31.5	0.004
	≥ 65 years	137	44.3	32.3	15.6	
Location	Central	204	45.0	35.7	19.4	0.001
	Peripheral	74	67.6	55.0	36.2	
Chemotherapy	No	64	45.0	33.0	19.9	0.026
	Yes	214	52.8	42.9	25.2	
Radiation pneumonitis	No	194	51.9	45.3	29.1	0.041
	Yes	84	48,8	30.7	14.7	
Short-term effects	PR	158	60.6	50.5	31.8	
	SD	82	47.6	37.8	20.5	< 0.001
	PD	38	21.1	7.9	0	
PFS1 subgroup	< 12 ms	98	34.7	25.4	8.4	< 0.001
	≥ 12 ms	180	62.1	49.2	34.1	

OS: overall survival; PD: progressive disease; PR: partial response; SD: stable disease.

**Table 3:** Cox Multivariate Analysis of OS

	HR (95.0% CI)	P value
Age		< 0.001
< 65 years	1 (ref)	
≥ 65 years	0.50 (0.371 - 0.674)	
Location		
Central	1 (ref)	0.002
Peripheral	1.74 (1.226 - 2.483)	
Radiation pneumonitis		0.048
No	1 (ref)	
Yes	0.736 (0.543 - 0.997)	
PFS1 subgroup		< 0.001
CR + PR	1 (ref)	
SD + PD	0.53 (0.396 - 0.709)	
PFS1 subgroup		< 0.001
< 12 ms	1 (ref)	
≥ 12 ms	2.70 (2.006 - 3.649)	

CI: confidence interval; HR: hazard ratio; CR: complete response; PD: progressive disease; PFS: progression-free survival; PR: partial response; SD: stable disease.

#### PFS univariate analysis

Smoking status (P = 0.005), alcohol consumption (P = 0.002), lesion location (P = 0.001), radiation pneumonitis (P = 0.045), actual completed radiotherapy dose (P = 0.017), and recent efficacy (P = 0.001) were associated with PFS (P < 0.05) (Table 4), while gender, age, ECOG score, chemotherapy status, family history of malignancy, pathological differentiation, T stage, N stage, and TNM stage were not associated with PFS. In multivariate Cox analysis, radiotherapy efficacy (P = 0.044) and actual completed radiotherapy dose (P = 0.020) were independent prognostic factors for PFS (P < 0.05) (Table 5). Patients who received radiotherapy and achieved CR or PR, with a radiotherapy dose ≥ 60 Gy, had longer PFS (P < 0.05).

**Table 4:** Single Factor Analysis of PFS1

Factor	Group	Case, n	PFS1 (%)			P value
			3 years	5 years	10 years	
Smoke	No	55	40.0	33.9	23.7	0.005
	Yes	223	25.9	18.7	4.1	
Alcohol	No	153	35.3	26.7	14.9	0.001
	Yes	125	20.3	15.6	1.5	
Location	Central	204	24.3	16.9	5.1	0.001
	Peripheral	74	41.9	35.0	16.7	
Actual completed dose of radiotherapy	< 60 Gy	81	24.6	20.3	3.9	0.017
	≥ 60 Gy	197	30.4	22.6	9.9	
Radiation pneumonitis	No	194	31.4	24.4	9.4	0.045
	Yes	84	22,6	16.2	5.5	
Stratification of first-line treatment efficacy	CR + PR	158	60.0	50.5	31.8	
	SD + PD	120	39.2	29.2	15.1	< 0.001

CR: complete response; PD: progressive disease; PFS: progression-free survival; PR: partial response; SD: stable disease.

**Table 5:** Cox Multivariate Analysis of PFS1

	HR (95.0% CI)	P value
Radiotherapy efficacy		0.044
CR + PR	1 (ref)	
SD + PD	0.756 (0.576 - 0.992)	
Actual completed dose of radiotherapy		0.020
< 60 Gy	1 (ref)	
≥ 60 Gy	1.388 (1.053 - 1.829)	

CI: confidence interval; CR: complete response; HR: hazard ratio; PD: progressive disease; PFS: progression-free survival; PR: partial response; SD: stable disease.

#### χ^2^ test

Patients receiving chemotherapy showed better recent response (CR + PR) (P < 0.05). Whether chemotherapy is associated with treatment failure patterns, patients who received chemotherapy had a lower incidence of distant metastasis (P < 0.05). On comparison of first-line treatment regimens, objective response rate (ORR: CR + PR): CCRT ± consolidation chemotherapy was received in 35 cases (71.4%), SCRT in eight cases (50%), monotherapy radiotherapy in 23 cases (36.5%), and induction chemotherapy + radiotherapy (chemotherapy)/CCRT in 92 cases (63.1%). The efficacy of CCRT ± consolidation chemotherapy was better (P < 0.05). In PFS stratification analysis, as of the follow-up date, patients with PFS ≥ 12 months had a lower risk of recurrence and metastasis compared to those with PFS < 12 months (P < 0.001).

## Discussion

Lung cancer is the most common malignant tumor, and patients often present with locally advanced or advanced disease at clinical presentation. Over the past three decades, CCRT has been the standard treatment for locally advanced NSCLC that is not operable, with a 5-year survival rate of only about 30%. In this study, the 1-, 3-, and 5- survival rates for 278 patients with locally advanced squamous cell lung cancer were 86.0%, 50.6%, and 40.0%, respectively, with a median survival of 37.8 months. The RTOG 0617 study showed that the 5-year survival rate of the standard dose radiotherapy and chemotherapy group was 23.0%, with a median survival time of 28.7 months [7]. The two fundamental oncologic treatment regimens for delivering the aforementioned combined modalities are: 1) sequential, whereby chemotherapy modality is completed prior to the initiation of the radiotherapy and 2) concurrent, according to which radiation and chemotherapy are administered simultaneously [8]. The former approach diminishes the risk of distant metastases, may also reduce the volume of the primary tumor making subsequent irradiation more effective, and may even make the tumor resectable. Nevertheless, prolonged total treatment time, postponed irradiation, and the possibility of accelerated repopulation of tumor cells can adversely affect local tumor control [9]. Until now, clinical trials assessing chemo-radiation in elderly patients (defined as older than 70 years) were performed by the Japan Clinical Oncology Group [10]. In this study, both OS and PFS were better than those in the aforementioned studies. The reason is that the study not only adopted the CCRT treatment model but also combined it with induction chemotherapy or consolidation chemotherapy, which increased the intensity of treatment and significantly improved patient survival. However, because it is a retrospective study, the chemotherapy regimen and the number of chemotherapy cycles are not uniform, and the chemotherapy regimen varies greatly with the changes of patients’ condition after progression, so only general comparative results can be obtained. The specific comparison of the number of induction chemotherapy cycles and chemotherapy regimens needs to be confirmed by prospective randomized controlled trials.

Due to the large size and wide range of tumors in locally advanced patients, induction chemotherapy can reduce the incidence of distant metastasis and shrink the tumor, thereby reducing the volume of the radiotherapy target area, improving the efficacy of radiotherapy, and alleviating its side effects. Therefore, CCRT after induction chemotherapy has gradually become a model for treating locally advanced NSCLC that is not operable. A meta-analysis of two studies of induction chemotherapy followed by CCRT versus CCRT alone and five studies of induction chemotherapy followed by CCRT versus CCRT followed by consolidation chemotherapy published in the same period was conducted [11]. Our results showed that there was significant benefit of induction chemotherapy plus CCRT compared to CCRT alone on 5-year OS without 1-, 2-, 3-, and 4-year OS. The analysis also indicated that induction chemotherapy was as effective as consolidation chemotherapy for patients who received CCRT on overall response and OS. Treatment-related toxicity was similar between the two group; however, leucopenia was significantly decreased in patients treated by induction chemotherapy (odds ratio (OR) = 0.43; 95% CI: 0.30 - 0.62; P < 0.00001). In conclusion, 5-year OS could be improved when induction chemotherapy was added into CCRT for patients of NSCLC. Except low rate of leucopenia, induction chemotherapy was no difference compared to consolidation chemotherapy in patients with NSCLC treated by CCRT. However, it is worth noting that toxicity was increased with CCRT compared with a sequential approach, with significantly higher rates of myelosuppression and esophagitis (18% grade 3/4 vs. 4% with sequential administration; P < 0.001) [12]. Fournel et al [13] studied 127 patients with stage III unresectable NSCLC, comparing induction chemotherapy + CCRT versus CCRT + consolidation chemotherapy. The median OS was 19.6 and 16.3 months, respectively, with 4-year survival rates of 21% and 30% (P = 0.2). Hematological and non-hematological toxicities were similar between the two groups, except for grade 3/4 esophagitis, which was more common in the consolidation group (17% vs. 10%). Local recurrence rates were 23% vs. 13%, and distant metastasis rates were both 29%. CCRT combined with consolidation chemotherapy is recommended. In our study, the short-term efficacy of CCRT ± consolidation chemotherapy was better, but there was no significant difference in 5- and 10-year survival rates. This may be related to retrospective studies, confounded factors, chemotherapy regimens, and unequal cycles. We look forward to future prospective randomized controlled trials for further analysis.

Analysis of survival data, treatment toxicities, and rates of treatment completion was performed for 241 patients who underwent chemoradiotherapy for unresectable stage III NSCLC within Leeds Cancer Centre from January 2011 to December 2014 [14]. Median survival was 18.8 months following SCRT compared to 22.7 months following CCRT (hazard ratio (HR) 0.90, 95% CI: 0.67 - 1.20, P = 0.46). Median follow-up was 21 months. The clinical benefit rate for CCRT compared to SCRT was 22.7% versus 24%. In the CCRT group, 63.8% patients completed treatment compared to 46% in the SCRT arm (P < 0.01). The 90-day mortality rates were low in CCRT and SCRT cohorts at 4.3% and 1%, respectively. There was greater pulmonary toxicity following CCRT versus SCRT (13.5% versus 1.0%, P < 0.01).

This retrospective study included stage III NSCLC patients aged ≥ 65 years, with ≥ 1 claim for systemic therapy (ST) or radiotherapy (RT) within 90 days of diagnosis, identified in SEER-Medicare data (2009 - 2014) [15]. Of 3,799 patients identified, 21.7% received ST; 26.3% received RT; and 52.0% received CCRT. CCRT patients tended to be younger (P < 0.001), White (P = 0.002), and have a good predicted performance status (P < 0.001). Patients who saw all three specialist types (medical oncologist, radiation oncologist, and surgeon) had increased odds of receiving CCRT (P < 0.001). ST and RT patients had higher mortality risk versus CCRT patients (HR (95% CI): ST: 1.38 (1.26 - 1.51); RT: 1.75 (1.61 - 1.91)); P < 0.001). Given the survival benefit of receiving CCRT over single-modality therapy, physicians should discuss treatment within a multidisciplinary team, and be encouraged to pursue CCRT for patients with unresectable stage III NSCLC.

While striving for better efficacy and longer survival, the side effects of radiotherapy and chemotherapy are also critical issues that need to be considered in anti-tumor treatments. Therefore, when administering anti-tumor therapy to patients, it is essential to balance benefits with tolerability. This is especially true for elder patients, who generally have poorer health conditions and multiple comorbidities, often leading to lower tolerance and compliance with anti-tumor treatments [16]; older and/or frail patients; those with cardiovascular or respiratory comorbidities in which treatment-related adverse events may be higher [17]. Chen et al analyzed 117 elderly patients with stage III and IV NSCLC, comparing the efficacy and toxic side effects of neoadjuvant CCRT with neoadjuvant radiotherapy alone. Only stage IV patients showed a survival benefit from CCRT (P = 0.012), while there was no significant difference in stage III patients (P = 0.055). In terms of hematological toxicity, the CRT group had significantly higher blood cell counts (35.0% vs. 0%; P < 0.001) and neutrophil counts (33.3% vs 0%; P < 0.001) compared to the RT group [18]. An early Japanese phase III trial aimed to compare the efficacy and safety of standard chest RT combined with or without low-dose carboplatin chemotherapy in elderly patients with locally advanced NSCLC. However, after enrolling 46 patients from November 1999 to February 2001, the trial was prematurely terminated due to the death of four patients (one in the RT group and three in the CRT group) attributed to treatment-related causes [19]. Although retrospective studies have shown that simultaneous chemoradiotherapy is as effective in elderly patients as in younger patients [20], the optimal regimen for chemoradiotherapy remains unclear [21].

In response to the unresolved chemotherapy regimen for elderly patients, some scholars have been continuously experimenting. A phase II study (LOGIK1902) evaluated the efficacy and safety of weekly carboplatin combined with radiotherapy in 37 elderly patients with stage III NSCLC. The ORR was 63.9%. The median PFS was 14.6 months, and the median OS was 25.5 months. One patient (2.8%) experienced grade 4 leukopenia, neutropenia, and thrombocytopenia. The study concluded that weekly carboplatin and concurrent radiotherapy were safe and effective for elderly patients with locally advanced NSCLC [22]. A meta-analysis compared the efficacy and toxicity of different CCRT regimens for treating locally advanced NSCLC. Relevant studies from PubMed, EMBASE, Web of Science, and CENTRAL were retrieved from October 1, 2020 onwards. This network meta-analysis included 22 studies involving 18 regimens, showing that the carboplatin + paclitaxel and pemetrexed + cisplatin regimens had better efficacy and lower toxicity. Therefore, it was concluded that CCRT (pemetrexed + cisplatin) and CCRT (carboplatin + paclitaxel) may be the best choices for treating locally advanced NSCLC [23].

Failure mode analysis was conducted. In our study, patients with multi-site lymph node metastasis at initial diagnosis had a shorter interval between PFS1 and PFS2 compared to those with single-site lymph node metastasis (P < 0.05), suggesting that multi-site lymph node metastasis is more prone to recurrence and metastasis. This aligns with the aforementioned research conclusions. Although comprehensive radiotherapy and chemotherapy can extend patients’ OS and PFS, in terms of failure mode analysis regarding recurrence and metastasis, whether it is the treatment modality (single radiotherapy or combined radiotherapy and chemotherapy) or the radiation dose (≥ 60 Gy or < 60 Gy), lung lesion progression, mediastinal, and supraclavicular lymph node metastasis are predominant (over 50%), with no statistically significant differences among subgroups. It may also be related to the imbalance in the patients we enrolled.

We are now in the era of immunotherapy. The landmark PACIFIC trial [24] reported a 5-year OS of 42.9% and a 5-year PFS of 33.1%. In our study, the 5- and 10-year OS rates for the overall cohort were 40.8% and 24.2%, respectively, and for the elderly subgroup, they were 32.3% and 15.6%, respectively. The 5- and 10-year PFS rates were 21.9% and 9.3% in the overall group, and 21.6% and 8.3% in the elderly group. The 5-year OS rate in our overall cohort was slightly lower than that in the PACIFIC study. It should be noted, however, that not all patients meet the eligibility criteria of the PACIFIC trial, particularly older or frail individuals with poor performance status who may not tolerate CCRT.

The single-arm, phase II, open-label PACIFIC-6 trial [25] included 120 patients with ECOG performance status (PS) ≤ 2, most of whom were aged ≥ 65 years, along with a small number of patients with WHO/ECOG PS 2. The median OS was 39.0 months, and the 3-year OS rate was 56.5%. Grade 3/4 adverse events (AEs) occurred in 27.4% of patients, and 6.0% experienced grade 3/4 potentially fatal or life-threatening AEs (PRAEs) during the treatment period. Pneumonitis was the most frequent PRAE, occurring in 17.1% of patients (any grade) and 1.7% (grade 3/4). There were three fatal AEs (2.6%), including one (0.9%) fatal PRAE due to pneumonitis. Additionally, 27.4% of patients discontinued durvalumab as a result of AEs from any cause. Although survival outcomes were improved, the toxicity profile remains a concern. Therefore, treatment strategies for elderly patients should be tailored with caution.

Given that elderly patients often have a poor clinical condition, comprehensive pre-radiotherapy evaluation and precise radiotherapy planning are particularly important. Mulita et al recruited 34 patients with histologically confirmed locally advanced NSCLC. All patients underwent 18F-FDG PET-CT-based RT simulation. Two sequential RT plans were created by the same radiation oncologist: one based on CT alone and the other PET-CT. 18F-FDG PET-CT detected distant metastases in 7/34 (20.6%) patients, altering the overall therapeutic plan in 4/34 (11.8%) and allowing radical RT in three of them who had oligometastatic disease (8.8%). It modified RT planning in 26/34 (76.5%) patients and clarified malignancy in atelectatic areas. Nodal involvement was identified in 3/34 patients (8.8%) and excluded in 3/34 cases, avoiding unnecessary nodal irradiation. Additional involved nodes were revealed in 12/34 (35.3%) patients, requiring dose escalation. Overall, changes to the tumor planning target volume (PTV) were made in 23/30 (76.6%) and to the nodal PTV in 19/30 (63.3%) cases (P < 0.0001). Primary tumor and nodal PTVs increased in 20/34 (66.7%) and 13/34 (43.3%), respectively [26].

In the current era of rapid technological progress, artificial intelligence has become indispensable in the diagnosis and management of lung cancer, serving to enhance diagnostic accuracy, revolutionize thoracic surgery by enhancing decision-making, improving patient outcomes, and guide optimal treatment [27].

### Conclusion

In conclusion, the prognosis of locally advanced squamous cell carcinoma of lung is poor, especially in elderly patients. If the general condition of the patient is good, it is recommended to receive radiotherapy with a dose of more than 60 Gy and try comprehensive treatment with tolerable toxic side effects.

## Data Availability

The authors declare that data supporting the findings of this study are available within the article.

## References

[1] Little AG, Gay EG, Gaspar LE, Stewart AK (2007). National survey of non-small cell lung cancer in the United States: epidemiology, pathology and patterns of care. Lung Cancer.

[2] Leivaditis V, Mulita F, Papatriantafyllou A, Grapatsas K, Koletsis E, Liolis E, Tasios K (2025). Unveiling the rare: an uncommon tale of synovial chondromatosis in an elderly patient's sternoclavicular joint. Kardiochir Torakochirurgia Pol.

[3] Leivaditis V, Mulita F, Liolis E, Nikolakopoulos K, Tasios K, Litsas D, Krinos N (2025). Unraveling the mystery of pleural calcifications: a diagnostic challenge in pleural disease. Arch Med Sci Atheroscler Dis.

[4] Leivaditis V, Baltagianni M, Liolis E, Baltayiannis N, Stanc G, Souka E, Batika P (2025). Inflammatory myofibroblastic tumor of the lung: a comprehensive narrative review of clinical and therapeutic insights. Kardiochir Torakochirurgia Pol.

[5] Hirsch FR, Scagliotti GV, Mulshine JL, Kwon R, Curran WJ, Wu YL, Paz-Ares L (2017). Lung cancer: current therapies and new targeted treatments. Lancet.

[6] Hung MS, Wu YF, Chen YC (2019). Efficacy of chemoradiotherapy versus radiation alone in patients with inoperable locally advanced non-small-cell lung cancer: A meta-analysis and systematic review. Medicine (Baltimore).

[7] Bradley JD, Hu C, Komaki RR, Masters GA, Blumenschein GR, Schild SE, Bogart JA (2020). Long-term results of NRG oncology RTOG 0617: standard- versus high-dose chemoradiotherapy with or without cetuximab for unresectable stage III non-small-cell lung cancer. J Clin Oncol.

[8] Rajappa S, Sharma S, Prasad K (2019). Unmet clinical need in the management of locally advanced unresectable lung cancer: treatment strategies to improve patient outcomes. Adv Ther.

[9] Alaswad M (2023). Locally advanced non-small cell lung cancer: current issues and recent trends. Rep Pract Oncol Radiother.

[10] Atagi S, Mizusawa J, Ishikura S, Takahashi T, Okamoto H, Tanaka H, Goto K (2018). Chemoradiotherapy in Elderly Patients With Non-Small-Cell Lung Cancer: Long-Term Follow-Up of a Randomized Trial (JCOG0301). Clin Lung Cancer.

[11] Luo H, Yu X, Liang N, Xie J, Deng G, Liu Q, Zhang J (2017). The effect of induction chemotherapy in patients with locally advanced nonsmall cell lung cancer who received chemoradiotherapy: A systematic review and meta-analysis. Medicine (Baltimore).

[12] Auperin A, Le Pechoux C, Rolland E, Curran WJ, Furuse K, Fournel P, Belderbos J (2010). Meta-analysis of concomitant versus sequential radiochemotherapy in locally advanced non-small-cell lung cancer. J Clin Oncol.

[13] Fournel P, Vergnenegre A, Robinet G, Lena H, Gervais R, Le Caer H, Souquet PJ (2016). Induction or consolidation chemotherapy for unresectable stage III non-small-cell lung cancer patients treated with concurrent chemoradiation: a randomised phase II trial GFPC - IFCT 02-01. Eur J Cancer.

[14] Spencer A, Williams J, Samuel R, Boon IS, Clarke K, Jain P (2021). Concurrent versus sequential chemoradiotherapy for unresectable locally advanced stage III non-small cell lung cancer: Retrospective analysis in a single United Kingdom cancer centre. Cancer Treat Res Commun.

[15] Bobbili P, Ryan K, DerSarkissian M, Dua A, Yee C, Duh MS, Gomez JE (2020). Predictors of chemoradiotherapy versus single modality therapy and overall survival among patients with unresectable, stage III non-small cell lung cancer. PLoS One.

[16] Hung A, Lee KM, Lynch JA, Li Y, Poonnen P, Efimova OV, Hintze BJ (2021). Chemoradiation treatment patterns among United States Veteran Health Administration patients with unresectable stage III non-small cell lung cancer. BMC Cancer.

[17] Bortolot M, Cortiula F, Fasola G, De Ruysscher D, Naidoo J, Hendriks LEL (2024). Treatment of unresectable stage III non-small cell lung cancer for patients who are under-represented in clinical trials. Cancer Treat Rev.

[18] Chen F, Hu P, Liang N, Xie J, Yu S, Tian T, Zhang J (2018). Concurrent chemoradiotherapy with weekly nedaplatin versus radiotherapy alone in elderly patients with non-small-cell lung cancer. Clin Transl Oncol.

[19] Atagi S, Kawahara M, Tamura T, Noda K, Watanabe K, Yokoyama A, Sugiura T (2005). Standard thoracic radiotherapy with or without concurrent daily low-dose carboplatin in elderly patients with locally advanced non-small cell lung cancer: a phase III trial of the Japan Clinical Oncology Group (JCOG9812). Jpn J Clin Oncol.

[20] Zaki M, Dominello M, Dyson G, Gadgeel S, Wozniak A, Miller S, Paximadis P (2017). Outcomes of elderly patients who receive combined modality therapy for locally advanced non-small-cell lung cancer. Clin Lung Cancer.

[21] Maggiore RJ, Zahrieh D, McMurray RP, Feliciano JL, Samson P, Mohindra P, Chen H (2021). Toxicity and survival outcomes in older adults receiving concurrent or sequential chemoradiation for stage III non-small cell lung cancer in Alliance trials (Alliance A151812). J Geriatr Oncol.

[22] Harada T, Sasaki T, Ishii H, Takemoto S, Hisamatsu Y, Saito H, Yoneshima Y (2024). A phase II study of weekly carboplatin and concurrent radiotherapy in older adults with locally advanced non-small cell lung cancer (LOGIK1902). Thorac Cancer.

[23] Zheng Q, Min S, Zhou Y (2022). A network meta-analysis for efficacies and toxicities of different concurrent chemoradiotherapy regimens in the treatment of locally advanced non-small cell lung cancer. BMC Cancer.

[24] Spigel DR, Faivre-Finn C, Gray JE, Vicente D, Planchard D, Paz-Ares L, Vansteenkiste JF (2022). Five-year survival outcomes from the PACIFIC Trial: durvalumab after chemoradiotherapy in stage III non-small-cell lung cancer. J Clin Oncol.

[25] Garassino MC, Khalifa J, Reck M, Chouaid C, Bischoff H, Reinmuth N, Cove-Smith L (2025). Durvalumab after sequential chemoradiotherapy in unresectable stage III non-small-cell lung cancer-final analysis from the phase II PACIFIC-6 trial. ESMO Open.

[26] Mulita A, Valsamaki P, Bekou E, Anevlavis S, Nanos C, Zisimopoulos A, Giatromanolaki A (2025). Benefits from (18)F-FDG PET-CT-based radiotherapy planning in stage III non-small-cell lung cancer: a prospective single-center study. Cancers (Basel).

[27] Leivaditis V, Maniatopoulos AA, Lausberg H, Mulita F, Papatriantafyllou A, Liolis E, Beltsios E (2025). Artificial intelligence in thoracic surgery: a review bridging innovation and clinical practice for the next generation of surgical care. J Clin Med.

